# On-chip sub-terahertz surface plasmon polariton transmission lines in CMOS

**DOI:** 10.1038/srep14853

**Published:** 2015-10-08

**Authors:** Yuan Liang, Hao Yu, Hao Chi Zhang, Chang Yang, Tie Jun Cui

**Affiliations:** 1 School of Electrical and Electronic Engineering, Nanyang Technological University, Singapore 639798; 2 School of Information Science and Engineering, and State Key Laboratory of Millimeter Waves, Southeast University, China 210018

## Abstract

A low-loss and low-crosstalk surface-wave transmission line (T-line) is demonstrated at sub-THz in CMOS. By introducing periodical sub-wavelength structures onto the metal transmission line, surface plasmon polaritons (SPP) are excited and propagate signals via a strongly localized surface wave. Two coupled SPP T-lines and two quasi-TEM T-lines are both fabricated on-chip, each with a separation distance of 2.4 μm using standard 65 nm CMOS technology. Measurement results show that the SPP T-lines achieve wideband reflection coefficient lower than −14 dB and crosstalk ratio better than −24 dB, which is 19 dB lower on average than the traditional T-lines from 220 GHz to 325 GHz. The demonstrated compact and wideband SPP T-lines have shown great potential for future realization of highly dense on-chip sub-THz communications in CMOS.

Future high performance computers require wideband on-chip communication between memory and microprocessor cores. The global interconnect by top-layer metal in present CMOS technology has limited bandwidth and large crosstalk ratio^1^,^2^. The lossy substrate with typical 10 Ω-cm resistivity introduces a low-impedance path between metal and substrate, resulting in narrow bandwidth and high loss. Moreover, at high operating frequency the current flow tends to crowd toward the surface of metal due to proximity effect, which leads to not only higher ohmic loss but also large electromagnetic coupling. As such, the CMOS metal based interconnect is not scalable to provide wide bandwidth for on-chip communication beyond tens of Gigabit per second (>10 Gbps) by one channel.

Though optical interconnects have shown great potential to replace electrical interconnects for high data throughput^3^,^4^,^5^, its source, transmission and detection are all difficult to be implemented in silicon. Terahertz (THz) bands have recently attracted great interest because all components can be realized in CMOS technology^6^,^7^,^8^,^9^. However, highly integrated on-chip high-speed interconnects by traditional transmission lines (T-lines) have critical limitations such as strong electromagnetic crosstalk between adjacent channels. Crosstalk neutralization techniques in presence by channel equalization consume high power and large silicon area with data rate limited not higher than 10 Gbps per channel^10^,^11^,^12^.

Due to the negative permittivity behavior^13^,^14^, surface plasmon polariton (SPP) is one special electromagnetic wave locally confined into the metal/dielectric interface, propagating in parallel to the interface with exponentially decaying in the direction perpendicular to the interface^15^,^16^,^17^,^18^. By introducing periodic sub-wavelength corrugation metal strips onto the T-line, SPPs can be established to propagate signals via strongly localized surface-wave in the metal/dielectric interface at frequency up to THz. Such a surface EM wave can be supported at low frequency region whose propagation adapts to the curvature or holes of the surface. Even though previous works have demonstrated the field-localization ability of SPP T-lines by both numerical and experimental approaches, their implementations are limited in microwave region on board level with bulky size and loss^19^,^20^,^21^,^22^,^23^,^24^,^25^,^26^. Moreover, the capability to strongly attenuate electromagnetic crosstalk in an intensively integrated environment by on-chip SPP T-line has not well been exposed or explored as far. In this paper, on-chip SPP T-line is investigated at the sub-THz region in standard CMOS process that shows great potential of system on chip integration with other components in CMOS for on-chip sub-THz communication.

The physical layout of the proposed structure is illustrated in Fig. 1. Two on-chip SPP T-lines are back-to-back placed to form a broadband low-loss, low-crosstalk coupler. Such a plasmonic metamaterial consists of a metal strip with thin film thickness, in which a 1D periodical array of grooves is drilled. The propagation of confined mode is adapted to the curvature of the surface, and the resulting crosstalk between the two back-to-back placed SPP T-lines will be significantly reduced. For comparison, two traditional quasi-TEM T-lines are also realized to form an on-chip coupler with line space of 2.4 μm in standard 65 nm CMOS process, which shows large loss and strong crosstalk at sub-THz. Measurement results show that the SPP T-lines achieve wideband reflection coefficient lower than −14 dB and the crosstalk ratio better than −24 dB, which is 19 dB lower on average than the traditional T-lines from 220 GHz to 325 GHz. While crosstalk is one of the greatest detrimental issues since it strongly distorts the signal integrity when placing multiple interconnects together at the high operation frequency, the compact and wideband SPP T-lines potentially replace the conventional on-chip T-line to meet the stringent requirement of future densely interconnect at high operation frequency for future high performance computer server.

## Result

### Excitation of on-chip SPP T-line

The surface plasmon polariton (SPP) propagating at the flat interface between a real metal and a dielectric are naturally 2D electromagnetic waves^27^,^28^,^29^. Confinement of EM wave is realized since the propagation constant is greater than the wave vector *k* within the dielectric, resulting in an evanescent decay on both sides of the interface. Essentially, because the SPP dispersion curve lies to the right of the light line of the dielectric (given by *ω* = *ck*)^29^, among optical applications the SPPs excited by 3D light beams is impossible unless special techniques for phase-matching are utilized^29^,^30^,^31^. Various optical techniques have been proposed to fulfill phase matching, including grating coupling and excitation with highly focused beams. Normally, for geometries exhibiting strong field-localization below the diffraction limit, the overlap between the excitation beam and the coupled SPP mode is small, leading to low excitation efficiency.

While those optical excitation schemes are suitable for the development of SPP propagation and functional plasmonic structures, in practice the SPPs used in the design of silicon integrated circuits will normally require high conversion efficiency (and hence large-bandwidth) coupling schemes. In on-chip electronic communication, the on-chip interconnects are generally connected to CMOS transistors or other passive devices whose bandwidths are commonly much narrower than that of optical devices. Given an electric excitation at one edge of SPP T-line, both momentum and impedance matching are essentially required to maintain high excitation efficiency. At the same time, the converter with exponentially grading CPW^19^ is difficult to be integrated on chip. As such, it is important to first investigate the impedance matching and the resulting reflection loss for the realization of on-chip SPP T-line.

In general, there are two dominative loss mechanisms contributing to the guiding of SPP T-lines: the return loss and the insertion loss (or transmission loss). While the insertion loss can be improved over other traditional T-line structures due to the confinement of EM energy at sub-THz (discussed in next section), the return loss begins to dominate as long as the periodical array decouples incident beam into the propagating SPPs. To understand how the mismatch affects the excitation, Fig. 2a shows the simulated input reflection coefficient (S_11_) of the designed on-chip SPP T-line as a function of groove depth *h*. To mimic on-chip interconnection, a plane TEM wave is injected from one edge of the structure while the other terminal is resistively terminated. As observed, the designed structure cannot maintain highly efficient coupling from 3–6 THz, which confirms the limited bandwidth of the low reflection region. Generally, the tight confinement can be achieved by merely increasing the groove depth *h* as long as the periodical grooves remain in deep subwavelength scale. However, the stronger field localization does not necessarily bring about the same amount of improvement for propagation if the coupling efficiency is taken into account: the stronger the confinement, the lower the bandwidth of efficient coupling. This comes from the fact that, owing to a considerably large momentum mismatch at the injection interface, only a minor fraction of the incident TEM wave can be converted to SPPs mode and coupled to the periodical grooves, leading to strong reflection of incident wave and energy loss. The return loss is maximized around the asymptote frequency, at which the propagation wave vector is large enough to cause significant momentum mismatch. Therefore, for the on-chip realization of SPP T-line, the operation frequency should be lower than the asymptote frequency.

### Design of on-chip SPP T-Line

**Figure 1:**
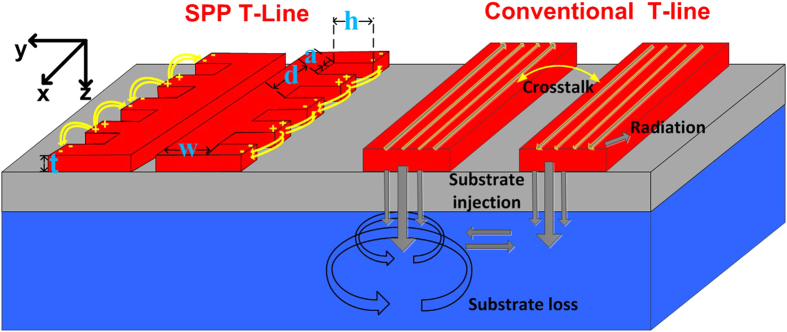
The layout and *E*-field distribution of the on-chip SPP/conventional T-line in lossy substrate environment, *d*, *h*, *a*, *w* denotes the periodic pitch, groove depth, groove width and line width of SPP T-line, respectively. The magnetic field of SPP T-line is directed to the *x* direction while the electrical field is guided by the grooves in the *y*-*z* plane.

Recall that for a compact SPP T-line structure with subwavelength lattices, the return loss (S_11_) is pretty high at several THz due to the low coupling efficiency of incident beam for the propagation of surface wave. It nevertheless appears lower than −10 dB for frequency below 1 THz, at the cost of weaker field-localization below the diffraction limit. In this case, the dispersion curves of propagating SPPs starts to bend with respect to the light line but the bending degree is lower than the case at asymptote frequency, indicating that parts of EM energy maintain their polarization characteristics after being injected into the structure. This scenario is quite commonly observed in CMOS technology since the substrate is highly conductive and the surface current can be supported following onto the surface of substrate, in a way to excite higher order modes. In fact, the typical features of SPP T-line can be evaluated through the dispersion diagram^19^,^20^,^21^, and the confinement of surface modes reaches its maxima at asymptote frequency. In the limit of metal thickness *t* →∞ neglecting diffraction effects and for λ ≫ *d*, the dispersion relationship can be approximated by 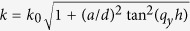
20, where *k* is the modal wave vector with k_0_ = 2π/λ, and the *y* component (see Fig. 1) of the wave vector is related with corresponding *x*, *z* component by 
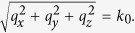
 The electromagnetic fields inside the periodical groves are independent of either *x* or *z* components for the mode polarization, leading to *q*_*y*_ ≈ *k*_0_. This implies that an asymptote appears for *k*_*0*_*h* = π/2, or equivalently, for a normalized frequency *d*/4*h*. The group velocity for energy propagation, given by the slope of the dispersion curves, is largest for excitation for this case, occurring in the first Brillouin zone (Re(k) = π/d). In contrast, the dispersion relation of conventional T-line features a constant slope with the modal wave vector, given by k = ω/c, which presents a highly delocalized nature at THz frequencies. For the design of plasmonic waveguide, normally a deep periodical groove is required, which is difficult for CMOS on-chip implementation as its physical dimension becomes unacceptably large with poor integration density. Instead, in what follows, for ease of impedance matching at the injection interface as well, we investigate the SPP property by designing a compact SPP T-line structure operating at frequencies that are lower than asymptote frequency. Upon this case, the reflection loss can be neglected, leaving the field localization ability of on-chip SPP T-line fully demonstrated.

**Figure 2:**
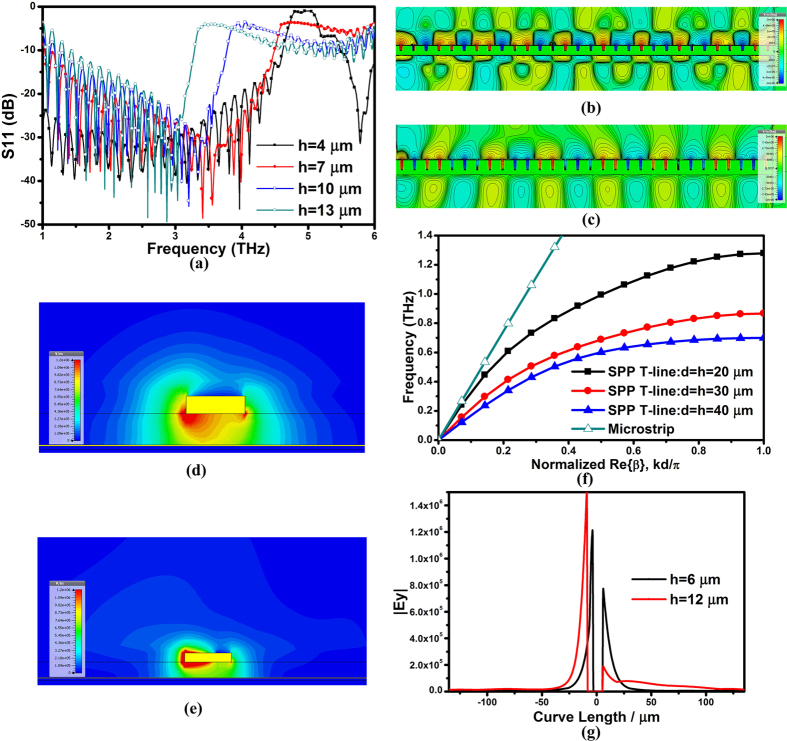
(**a**) Simulated input reflection coefficient (S_11_) of the designed on-chip SPP T-line for different groove depth *h* with *d* = 15 μm, *a* = 2.4 μm, *w* = 5 μm, (**b,c**) The simulated amplitude of *E*-field distribution of the designed SPP T-line (a: *h* = 6 μm, b: *h* = 12 μm) evaluated at the *xy* plane using CMOS process, (**d,e**) *E*-field distribution on the cross-section of the corrugated metal strip: *h* = 6 μm, d: *h* = 12 μm) at *yz* plane, also at 3 THz, and (**f**) The simulated dispersion diagram with different periodic pitch *d* and groove depth *h* ranged from 20 μm to 40 μm. The dispersion diagrams of conventional on-chip T-line are also plotted for comparison. (**g**) *E*-field enhancement along the vertical cut for *h* = 6 μm and *h* = 12 μm, respectively.

The CMOS-based SPP T-line structure is designed as shown in Fig. 1. The periodic pitch *d* is chosen to be 15 μm which is far less than the operation wavelength, and the line width *w* is 5 μm. Noted that the conventional microstrip line with *w* = 5 μm has a characteristic impedance of around 50 Ω from 10 GHz to 3 THz in this process. The SPP modes are generated in the structured line, as can be confirmed by the full-wave near-field simulation using finite-difference-time-domain (FDTD) method embedded in commercial EM simulator CST Studio. The boundary condition is set as open to simulate the real space. The boundaries are at large distances from the metal structure as well to avoid significant reflection. With the surface mode confined by the corrugated strip, we are now in a position to evaluate the *E*-field distribution of the guiding property. Figure 2b,c illustrate the simulated *E*-fields (*E*_*x*_ components) evaluated at the top of corrugated strips with different corrugations depth at 3 THz. The color-scale from red (the highest amplitude) to blue (the lowest amplitude) indicates the tight confinement of EM wave within the comb-shaped metal strips. In this experiment, instead of defining a monopole excitation, the TEM wave is injected from one edge of SPP T-line with the other terminal loaded with 50 Ω impedances. Note that this excitation configuration is more akin to the signal injection manner when the SPP T-lines are physically connected to other on-chip devices. Due to a non-perfect transition from the TEM wave to the surface wave at the injection interface, the excited surface mode is not completely restricted within the grooves. However, one can still observe that part of the EM field is strongly localized within the comb-shaped metal strips and decays at the metal/dielectric interface. We observe that the surface EM waves are tightly confined and propagate along the SPP T-line with small losses at 3 THz as well. In addition, the confinement of surface mode can be effectively enhanced by solely increasing the groove depth *h*, which can be confirmed by the observation of corresponding *E*-field distribution shown in Fig. 2d,e evaluated at the *yz* plane. Different from the board level implementation at low frequencies, a ground plane realized by the bottom copper metal M1 is required here to ensure the *E*-field to be mainly restricted between the two conductors. Without the ground, a great portion of energy will be absorbed by the lossy substrate, and the resulting field confinement by the designed structure becomes less effective. In silicon process, the propagating modes are not only attenuated due to absorption by the interlayer dielectrics, but also from re-radiation into the higher-index silicon substrate. Again, the dispersion diagram is re-simulated with the consideration of ground. It shows that the asymptotic frequency is still clearly bending away from the light line except that it slightly increases. Such a dispersion relation is further examined by varying the groove depth *h* as shown in Fig. 2f. Clearly, the asymptotic frequency decreases with deeper the grooves, in consistence with the case without ground beneath the SPP T-line. Specifically, when both the periodic pitch *d* and groove depth *h* are equal to 40 μm, the asymptotic frequency is down to approximate 0.6 THz, presenting stronger confinement toward sub-THz region. As such, the ground plane inherently has negligible influence on the SPP property while it helps to further reduce radiation loss. The dispersion relation of conventional on-chip T-line with underneath metal ground is illustrated in Fig. 2f as well for comparison, which shows a linear relationship with the modal wave vector, implying highly delocalized feature in the range of THz frequencies. On the other hand, the details of confinement can be clearly observed from *E*-field enhancement in the cross-sections perpendicular to the strips, as illustrated in Fig. 2g. The field clearly decays exponentially along the orthogonally lateral *y* direction, illustrating the typical feature of SPP mode, while the decay becomes stronger as *h* increases. In summary, all of the above studies reveal the feasibility of confining surface wave by the periodical sub-wavelength grooves structured T-line in standard CMOS technology.

**Figure 3:**
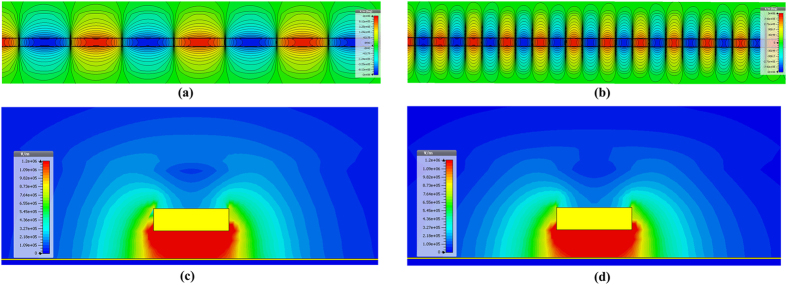
(**a,b**) The simulated amplitude of *E*-field distribution of the conventional transmission line evaluated at the *xy* plane using the same process at 1 THz and 3 THz, respectively, and (**c,d**) *E*- field distribution on the cross-section of the flat metal strip (*yz* plane) also at 1 THz and 3 THz, respectively. The width of T-line is 11 μm with wideband impedance matching up to above 3 THz. A ground plane realized by the bottom copper layer (M_1_) is implemented as an *E*-field shielding layer.

In contrast to TM polarization in SPP T-line, the conventional transmission line propagates TEM mode along the structure. As shown in Fig. 3a,b, the current intensity of the microstrip line tends to crowd on the metal surface. Record that in such a scenario the effective metal resistance will increase and therefore degrades the signal transmission. Given that the *E*-field is now dominating near the flat metal surface, more energy would be radiated out in form of radiation loss and crosstalk to adjacent conductors. Even though most of the *E*-field can be restricted to the metal surfaces between T-line and underlying ground as illustrated in *E*-field distribution at the *yz* plane, shown in Fig. 3c,d, the radiation outside of T-line remains strong, leading to more energy penetration into the dielectric medium and hence results in larger radiation loss. Upon this case, the ground inherently introduces additional loop path to form electromagnetic crosstalk to the nearby conductors through the current flowing on the ground surface, which will be even worse as frequency rises up. To be more specific, considering that the on-chip T-line propagation constant is closed to *k*_*0*_ at the light line, the EM wave extends multiples of wavelengths into the dielectric space. As frequency increases, such extension basically becomes stronger, leading to more radiation absorbed by the lossy medium as shown in Fig. 3a,b. More importantly, the radiation loss cannot be improved by simply changing the dimension of T-line. All these features manifest T-line an unsuitable candidate as on-chip interconnects in high speed THz wireline systems.

### Wideband low-loss on-chip transmission

**Figure 4:**
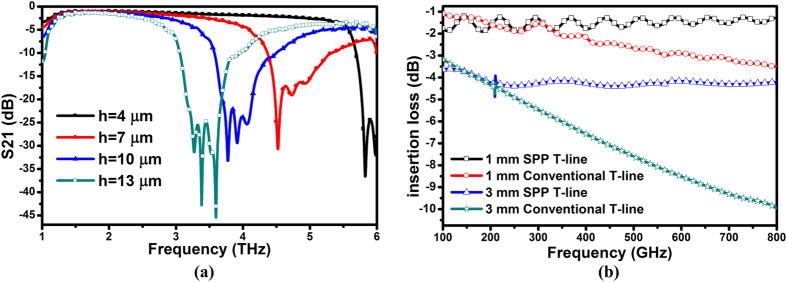
(**a**) Simulated transmission coefficient (or insertion loss S_21_) of the SPP guided wave as a function of the groove depth *h* with *a* = 2.4 μm, *w* = 5 μm, *d* = 15 μm, (**b**) comparison of simulated insertion loss for both SPP T-line and convention T-line with different length.

Next, we examine the transmission property of on-chip SPP T-line with comparison to conventional on-chip T-line structure. While the bandwidth of conventional T-line is greatly affected by the highly conductive substrate at THz, the field localization of surface plasmonic in SPP T-line can be easily governed by metal geometry. To verify this, we begin by examining the spectral transmission properties of designed SPP T-line up to several THz. In Fig. 4a, we show the simulated wideband transmission spectra with *h* = 4, 7, 10 and 13 μm. There are a few of notable characteristics in these spectra. As observed, when the grooves are very shallow (i.e. 7 μm deep or less), despite of an obvious high-frequency cutoff, the SPP T-line transmission spectra appear to be relatively broadband. As the groove depth begins to increase, both the bandwidth and cut-off frequency of this resonance decrease. As the groove depth further increases, the single wideband transmission resonance is replaced by multiple narrowband transmission resonances. In the theoretical limit of transmission by guiding SPPs, there are no modes appearing at frequencies above the Bragg frequency *f*_*B*_ = *c*/2*d*, where *c* is the speed of light. Here, the anti-resonance (AR) frequency, which is used to characterize the frequency corresponding to the signal being sharply attenuated, can be applied to explain the transmission properties of the periodical curvature structures due to Fano-interference phenomenon^32^. Even so, the transmission maintains low loss and wideband below the AR frequency.

We then compare the transmission for both on-chip T-line structures at frequencies less than 1 THz, where the advance THz integrated circuits normally operate. Here, a gradient groove structure is required at both edges of SPP T-line to reduce the reflection loss while maintains a necessary momentum matching at respective injection interface. With low return loss, the loss of SPP T-line is mainly attributed to the metal resistive loss at sub-THz. Figure 4b shows the comparison results. Clearly, the transmission of on-chip SPP T-line has very wide bandwidth with low loss, whereas the conventional T-line suffers from strong attenuation in sub-THz. This observation confirms the insensitivity of surface wave to the low-resistive substrate profile. Specifically, the insertion loss of 3 mm long SPP T-line is almost 3 times to that of its 1 mm counterpart across a very wide band, akin to simply cascading of three 1 mm unit-cells. However, the 3 mm long traditional T-line suffers from much larger attenuation than the loss added by three 1 mm unit-cells, illustrating its vulnerability to lossy substrate. As a result, by properly structuring the top metal, the loss of SPP T-line can be minimized across wideband, which cannot be achieved by a bare T-line in CMOS at sub-THz.

Further observation of Fig. 4b implies a vanishing of superiority on transmission by SPP T-line regardless of trace length, revealing another feature of surface mode adapted to the periodical sub-wavelength grooves: localization decreases for frequencies lower than 200 GHz (ω ≪ ω_p_), where *β* → *k*_*0*_ due to their large free electron density. In addition, the large complex permittivity leads to negligible field penetration into the conductor and thus delocalized fields. SPPs at these low frequencies therefore resemble a homogeneous light field in air incident under a grazing angle to the interface, and are also referred to *Sommerfeld-Zenneck* waves^33^. As such, the SPP T-line behaves more akin to a bare T-line at low frequencies.

**Figure 5:**
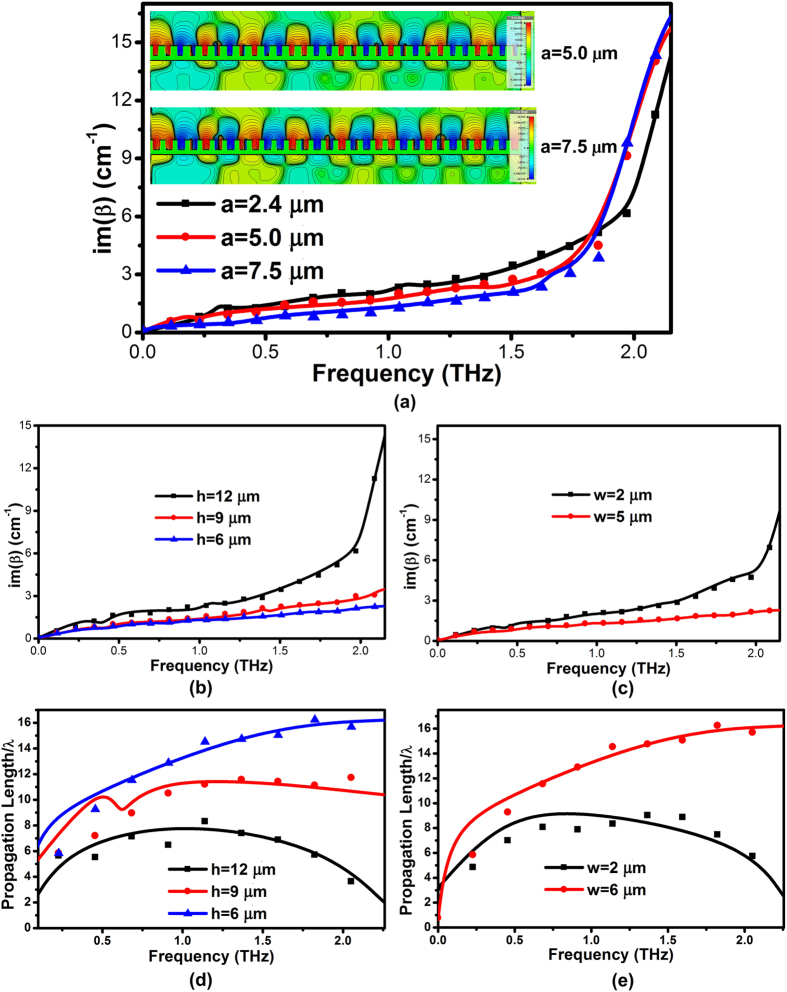
(**a–c**) The imaginary part of SPP modal wave vector *k* for various (**a**) groove width *a*, (**b**) groove depth *h* and (**c**) line width *w*, respectively. The insect in (**a**): *E*-field distribution of SPP T-line evaluated at the *yz* plane for various groove width *a*. (**d**,**e**) The normalized propagation lengths of SPP T-line with different geometries: (**d**) various *h* = 6 μm, 9 μm and 12 μm, respectively, and (**e**) various *w* = 2 μm and 5 μm, respectively.

Note that the excitation of conducting electrons in a real metal inherently has interband damping^28^. As such, *ε*_*m*_ becomes complex, along with the SPP propagation constant 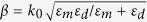. The SPP mode traveling along the structure therefore suffers from a damping factor caused by the imaginary part of *β*, leading to an energy attenuation length, which is also referred to propagation length given by *L* = (2*im*(*β*))^−1^, where *im*(*β*) is the imaginary part of the propagation vector of the bound modes. It represents the modal absorption attenuation suffered by the travelling coupled SPP wave along the structure. Figure 5a–c illustrate the absorption loss of on-chip SPP T-line up to THz. We observe that a decrease of groove width *a* leads to a marginal increase of modal loss, whereas a small increase of groove depth *h* results in a significant boost of loss. For instance, the absorption loss for the case with *h* = 12 μm is almost 7 times larger than the case with *h* = 6 μm. In addition, the losses of SPP T-line do not grow greatly with frequency unless it approaches the asymptote frequency. This implies physically that the absorption loss of SPPs is larger when the fields are more strongly confined to the wire. Evidently, the field confinement increases with frequency, as shown in Fig. 2f for various *h*. Figure 5a insets show the *E*-field distribution evaluated at the *yz* plane of SPP T-line for different *a* (the case for *a* = 2.4 μm is given in Fig. 2c), presenting a weaker field confinement for a larger *a*. Noted from Fano-interference as mentioned before, such interference between confined modes propagating in the forward and backward *z*-direction results in resonation, leading to the establishment of surface modes due to interactional couplings between cavity modes localized in individual grooves. As the cavity is gradually widened, such interference becomes diluted, in a way to release the modes coupling effect inside the subwavelength grooves. Thus, the field of confined modes are less efficiently excited for the case with larger *a*. As a result, the weaker field penetration into the metal/dielectric interface corresponds to a smaller energy dissipation, resulting in a lower loss for SPP T-line with larger *a*. The dependence of SPP modes absorption loss on the line width *w* is plotted in Fig. 5c while maintaining *w* + *h* constant. It indicates that simply widening the line width can effectively decrease the loss of SPP T-line.

Figure 5d extracts the normalized propagation length with different depth of groove *h* across a wide spectrum by numerical simulation. As observed, the achievement of increase in propagation length is accompanied by reducing groove depth *h*, akin to a simultaneous loss in confinement, as the mode gradually evolves into the TEM mode of the structure for vanishing groove depth. The SPP modes are now extending over multiple wavelengths into the dielectric medium as its confinement decreases when groove depth decreases. This property of SPP mode serves as a straightforward demonstration of a general principle of *tradeoff* between field-localization and propagation loss commonly observed in surface plasmon waveguides. Given that tight field confinement to the metal/dielectric interfaces inherently implies a considerable portion of the total mode energy localized inside the metal strip itself, the propagation length decreases due to Ohmic loss. Thus, as shown in Fig. 5d for various groove depths, guiding of electromagnetic energy with subwavelength mode confinement will demonstrate millimeter propagation length (e.g. about 9 mm at 300 GHz for the case *h* = 6 μm and *w* = 5 μm) that is sufficiently long for most on-chip communication. As expected, the propagation length is much more sensitive to frequency for deep grooves, because the Ohmic loss in the real metal becomes more significant with increasing frequency especially in the CMOS process. However, we observe that the propagation length of the bound mode can be greatly enhanced by simply reducing the groove depth *h* or increasing the line width *w*, as shown in Fig. 5d,e, respectively.

### On-chip crosstalk reduction

Crosstalk arises from the interaction of EM field generated by adjacent signals when they propagate through T-lines by mutual inductance *L*_*m*_ (magnetic field) and mutual capacitance *C*_*m*_ (*E*-field), which cause signal distortion and also loss. In existing CMOS technology, additional coupling mechanism such as substrate coupling, which is mainly due to the low resistivity substrate (10–20 Ω-cm) and high capacitance ratio (*C*_*sub*_, the magnitude of capacitance per unit thickness of dielectric), makes the electromagnetic isolation between two neighboring conductors difficult. Even though existing equalization techniques can marginally alleviate the channel crosstalk^10^,^11^,^12^,^34^,^35^,^36^, their communication data rate is limited not higher than 10 Gbps per channel. Other transmission line structures are presented^22^,^23^,^24^,^25^,^37^,^38^,^39^, but their applications are limited in board level with operation frequency lower than 20 GHz.

Owing to the advantage of governing the propagation property of EM energy by field-localization, interconnects realized by SPP T-line are expected to demonstrate less crosstalk. Recall that for a guided wave structure, the energy associated with the guided mode is confined near the metal surface, with the field decaying evanescently within the apertures. While the surface mode is restricted only to periodical grooves, electromagnetic coupling to both substrate and adjacent conductors can be strongly suppressed. Previous works were implemented in board level and their working frequency is limited in microwave region^26^,^27^,^28^,^29^.

**Figure 6:**
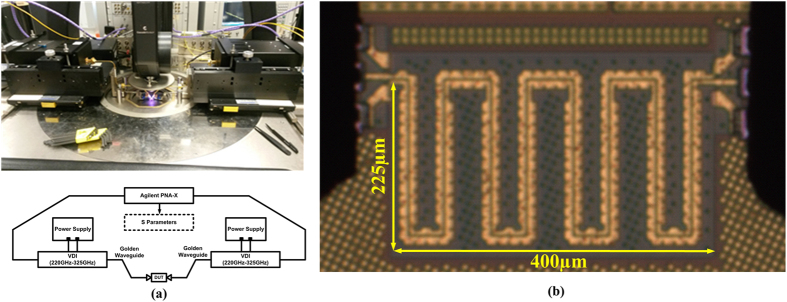
(**a**) Measurement setup for the THz SPP T-line coupler, (**b**) die micrograph of the SPP-based coupler in 65 nm CMOS for Terahertzes applications.

**Figure 7:**
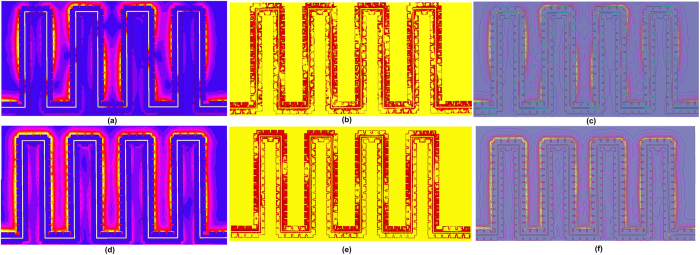
Near field simulation of proposed back-to-back placed on-chip SPP T-line coupler in 65 nm CMOS technology. The upper SPP T-line is the aggressor with EM field excitation at left-handed side terminal with the right-hand side termination loaded with 50 Ω impedances, and the lower SPP T-line is the victim trace with both ends terminated by 50 Ω impedances: (**a,d**) the simulated electrical-field distribution on the cross-section of SPP T-line coupler at 300 GHz and 500 GHz, respectively; (**b,e**) the simulated magnitude of body current density (*J*_*vol*_) distribution along the two SPP T-lines at 300 GHz, 500 GHz, respectively; (**c,f**) the simulated *E*-field lines distribution on the cross-section of SPP T-line coupler 300 GHz and 500 GHz.

**Figure 8:**
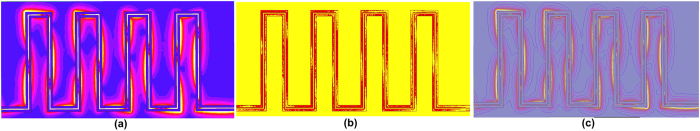
The simulated electrical-field distribution on the cross-section of conventional on-chip T-line coupler in 65 nm CMOS technology at 300 GHz, and the width of T-line is 5 μm while the lines spacing is 2.4 μm as well. The excitation configuration is the same as SPP T-line coupler; (**b**) the simulated magnitude of body current density (*J*_*vol*_) distribution along the two conventional on-chip T-lines at 300 GHz, and (**c**) the simulated *E*-field lines distribution on the cross-section of conventional on-chip T-line coupler at 300 GHz.

To evaluate the crosstalk reduction at sub-THz, two on-chip SPP T-lines are back-to-back placed closely to form an on-chip SPP-based coupler as shown in Fig. 6. Here, we call it coupler for simplicity while it may be used for intentional coupling in some circuit designs. Both SPP T-lines are designed with meandered shape due to limited area during this tapeout, but a long coupling length is necessary for intentionally strong coupling. The equivalent coupling lengths are 2 mm. All physical dimensions are as same as before with wire spacing of 2.4 μm. Conceptually, considering that the two SPP T-lines both have TM mode propagation along the signal trace while the EM energy is excited with surface wave and confined tightly within their respectively grooves, electromagnetic interference between adjacent conductor traces can be attenuated if the two SPP T-lines are back-to-back placed. In the simulation below, only one SPP T-line is excited as aggressor, while the adjacent SPP T-line is terminated by 50 Ω impedances at both edges as victim trace. The electromagnetic interference can be readily observed by near-field simulation shown in Fig. 7. Here, Fig. 7a shows the *E*-field distribution of the proposed SPP T-line coupler at 300 GHz. As predicted, even though two conductors are now closely placed, the SPP T-line can maintain its ability to confine surface waves with a small portion of their field penetrating into the adjacent conductor. Figure 7b shows the resulting current density distribution of the proposed SPP T-line coupler. Clearly, the excited SPP T-line conducts the majority of current along the structure, while only a small amount of current can be supported by the victim SPP T-line. This scenario can be further confirmed by Fig. 7c in which the *E*-field distribution is presented. As observed, the *E*-field can be locally confined by the subwavelength periodical grooves; and the *E*-field leakage into the adjacent conductor is weak. On the other hand, owing to the incomplete excitation of surface mode by the structure at this frequency, partial *E*-field interference between the two SPP T-lines is expected. For this reason, Fig. 7d–f further illustrates the *E*-field and current distribution of the proposed coupler at 500 GHz, implying an even more highly attenuated *E*-field interference between the two SPP T-lines. Here, the surface mode is obviously excited and more restricted within the grooves, and the resulting *E*-field is tightly confined as well. In this scenario, the propagation constant is much larger than ω/*c*, leading to an increase of wave vector with the dispersion curve bending more strongly away from the light line. The group velocity is reduced while the spatial extension of the mode along the corrugations decreases as well, leading to stronger confinement of surface wave into the periodical grooves, as indicated in Fig. 2b–g. Since most of the EM energy is now guided by surface mode at this frequency, the *E*-field interference between two conductor traces is thereby substantially attenuated. This phenomenon is in contrast to the T-line coupler, along which the TEM wave is propagated. As a comparison, a T-line coupler with identical meandered shape is designed as well, and the trace width and gap is 5 μm and 2.4 μm, respectively. The excitation configuration is the same as the case of SPP T-line coupler. Figure 8a shows the near-field simulation of T-line coupler at 300 GHz. By comparing to the SPP T-line coupler, the aggressor trace of T-line coupler induces much stronger crosstalk to victim trace. As a result, the victim is strongly excited to conduct more current while the propagation of aggressor is attenuated. As shown in Fig. 8c, the EM wave is not bound to flat metal surface, whose extension beyond metals basically becomes stronger with frequency, in a way to further enhance electromagnetic interferences. As such, to reduce crosstalk in sub-THz region by conventional on-chip T-lines, the metal separation needs to be increased, which in turn violates the strict requirement of highly dense wiring for future high performance servers.

### Measurement results of on-chip SPP T-line coupler

**Figure 9:**
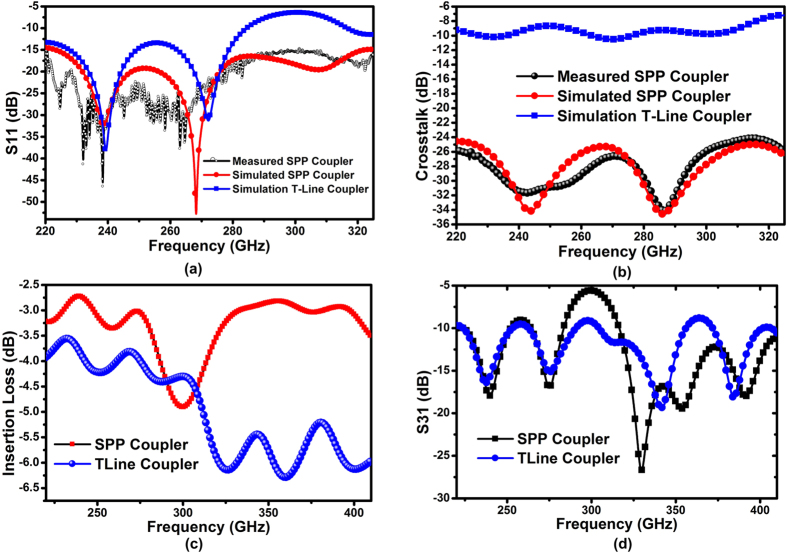
Measured and fitting *S* parameter results: (a) the measured and simulated results of the input reflection coefficient (S_11_) for the SPP coupler, and the simulated S_11_ of the T-line coupler. The *S* parameter extraction for T-line coupler is performed after the parameters fitting is done, (**b**) the measured and simulated result of crosstalk (S_41_) for the SPP coupler, and the simulation result for the conventional T-line coupler as a comparison, (**c**) the simulated insertion loss (S_21_) for both SPP/T-line couplers, and (**d**) the simulated near-ended coupling (S_31_) for both SPP/T-line coupler.

To demonstrate the superior performance in crosstalk reduction by the proposed SPP T-line coupler, the measured *S*-parameters are shown in Fig. 9. First of all, to have a fair comparison with the directional coupler realized by conventional T-line, the simulation results of the SPP coupler are fit to the measurement ones (S_11_ and S_41_). After the parameter fitting is done, the conventional T-line coupler is simulated using the same substrate profile. The T-line coupler also has a meander shape that resembles the structure shown in Fig. 6b. The measured and simulated reflection coefficients (S_11_) are shown in Fig. 9a with comparison to the T-line coupler. The measured S_11_ is below −14 dB over 220–325 GHz, showing wideband low reflection loss. The fitting of S_11_ is also presented (red curve), which only has a small deviation from the measurement result. As a comparison, the reflection loss of traditional T-line coupler becomes large (almost −5 dB) in the vicinity of 300 GHz, resulting in a power reflection of over 30% of the electromagnetic waves. Considering that T-line is naturally wideband, the reason for impedance mismatches is due to the strong coupling, which is further verified by Fig. 9b. As shown, the measured crosstalk (S_41_) of the proposed structure is lower than −24 dB which is averagely 19 dB better than that of the traditional T-line coupler within 220–325 GHz, illustrating great improvement in crosstalk reduction. In addition, the crosstalk is not obviously degraded as the frequency goes up while it becomes significantly worse for traditional T-line coupler, implying wider bandwidth achieved by the SPP coupler. For example, the crosstalk is only −25 dB obtained by the SPP coupler at 325 GHz but it rises to −7 dB for the T-line counterpart. This means that about 20% of the EM energy is coupled to the victim in the traditional T-line coupler; but only 0.3% of the energy leaks to the victim in the SPP-based coupler. Such a comparison by the measured *S* parameter can be confirmed by the *E*-field simulation shown in Fig. 7 and 8, all conducted in sub-THz region. The simulated crosstalk of SPP coupler is provided in the same figure as well, showing a good agreement with the measurement result. The small discrepancy may be due to the 10% dummy fill or process variation that have not been taken into account during the simulation setup. The insertion loss (S_21_) of both couplers is further simulated as shown in Fig. 9c. The proposed on-chip SPP coupler has 3 dB improvements in transmission coefficient over the traditional T-line as frequency beyond 300 GHz. There is a transmission valley in 270 GHz possibly due to the resonances between meandered wires. Figure 9d shows the simulated near-ended coupling (S_31_) for both structures. A hump is found at 300 GHz for the SPP coupler, which coincides with the S_21_ valley at Fig. 9c. However, the S_31_ remains lower than −10 dB for most frequencies across wideband, indicating low near-ended coupling. Note that in this design, the line spacing is 2.4 μm with attempt only to show the great crosstalk improvement obtained by the proposed SPP T-line coupler. During the circuit design phase this spacing is not necessary to be such narrow.

## Discussion

Recent state-of-art on-chip implementations of interconnects using conventional transmission line exhibit great attenuation and crosstalk at sub-THz, which in turn demand complex equalization techniques that consume considerably large power and silicon area. For instance, an interconnect model by assuming the Manhattan routing style in 0.18 μm CMOS technology was reported^40^. This model employs metal 5 as interconnect to construct BUS with 10-mm length onto lossy substrate. It shows that, due to strong coupling between neighbor conductors the transfer function |H| drops to below −150 dB for all loadings at frequencies higher than 100 GHz, inhibiting most applications of sub-THz on-chip communications. At the same time, the crosstalk in multi-channel series link is quantized for 10-mm interconnect in 0.13 μm CMOS^41^, in which both the adjacent crosstalk-to-signal ratio (ACSR) and distant crosstalk-to-signal ratio (DCSR) dramatically increase as frequency goes up. This tendency is apparently caused by the coupling capacitance that is inversely proportional to the spacing. All the above observation again confirms the significant crosstalk induced by TEM mode propagation along the long T-line. While the lower metal layers can partially alleviate the crosstalk (the thickness of lower metal layers are over 2–3 times smaller than that of the top copper metal, and the resulting coupling capacitance is reduced), the resistive loss will be proportionally higher^42^. Furthermore, as the case of commonly used backplane channels^43^, silicon carrier channels have very limited channel bandwidth, so the silicon carrier channel presents higher losses at higher frequencies. As a result, when high-frequency data is transmitted over long carrier channels, channel losses and limited channel bandwidth make the recovery of received data difficult. In sum, all these studies have shown great difficulty of realizing efficiently high speed data communication by conventional on-chip T-line.

To overcome all these fundamental limits by conventional transmission line at sub-THz, an on-chip surface plasmons polariton (SPP) T-line with sub-wavelength comb-shape onto the metal strip is demonstrated in sub-THz by CMOS technology. The dispersion characteristics and field distribution are investigated from the design perspective towards on-chip THz communication. It shows that such a structure can tightly confine the surface wave at the metal/dielectric interface, resulting in less loss with much smaller crosstalk. A SPP-based coupler is further fabricated in standard 65 nm CMOS with observation of great improvement on crosstalk reduction over wideband for on-chip interconnection. Measurement results show that the SPP coupler can guide the surface wave with lower than −15 dB reflection coefficient while the crosstalk improvement is averagely 19 dB across 220–325 GHz, which leads to nearly 3 dB lower transmission loss compared to the traditional on-chip T-line based design. Therefore, such a SPP T-line is very promising in the highly dense on-chip communication at sub-THz in CMOS technology.

## Methods

### On-chip implementation of SPP T-line coupler

To verify above design observations, a SPP T-line coupler was fabricated by 1P9M bulk 65 nm CMOS process with the die micrograph shown in Fig. 6b. The silicon area occupation is 400 μm × 225 μm. Due to limited silicon area, the proposed coupler is designed with a meander shape such that the equivalently physical length of the coupler is approximated 2 mm to provide a long coupling length. The spacing between  the two SPP T-line is 2.4 μm which is the minimum metal gap allowed by the according design rule. Such a close metal spacing is supposed to create a strong coupling for the long coupler, which would provide us a straight insight to evaluate the crosstalk reduction by the proposed structure. The top most copper layer (Metal 8) with thickness of 3.3 μm is exclusively employed for the design of a low-loss coupler, and the top Aluminum layer (Metal 9) with 1.325 μm thickness is used to form the Ground-Signal-Ground (GSG) Pad which has measured characteristic impedance around 50 Ω across the 220–325 GHz frequency range. Note that the thickness of metal has very limited influence on the dispersion relation of SPPs because the sub-wavelength nature still maintains (the *H*-field remains unquantized in the *x* direction.)^21^, while the resistive loss can be much improved by a thicker metal. It is important to know that, for SPP T-line the resistive loss (Ohmic loss) cannot be reduced by better EM field confinement. Two terminals of the coupler are directly connected to the signal trace of Pads and the other two ports are terminated by P + polysilicon resistors that were simulated having 50 Ω impedances from DC to 350 GHz. A lumped-element model was created to fit the measurement result of Pad over wideband, which is also an important part of the coupler design. Considering the Pad as part of the design, the SPP-based coupler is designed and optimized for the best crosstalk reduction before fabrication. The fabricated on-chip SPP-based coupler has the following design geometries: the periodic pitch *d* = 15 μm, groove width *a* = 2.4 μm, line width *w* = 5 μm, and the groove depth *h* = 6 μm. Their geometry meanings are presented in Fig. 1.

### Measurement setup for on-chip SPP T-line coupler

The capability of confining EM fields and crosstalk reduction can be evaluated by measuring the reflection coefficient S_11_ and the crosstalk S_41_. The THz signal is injected from one port into the SPP coupler, and the resulting S_41_ will present the coupling. The SPP interconnect is measured on CASCADE Microtech Elite-300 probe station and Agilent PNA-X (N5247A) with the VDI providing signal source from 220–325 GHz. Connectors, probe, waveguides and cable loss are well calibrated before on-wafer probe testing. The measurement setup is shown in Fig. 6a.

## Additional Information

**How to cite this article**: Liang, Y. *et al*. On-chip sub-terahertz surface plasmon polariton transmission lines in CMOS. *Sci. Rep*. **5**, 14853; doi: 10.1038/srep14853 (2015).
